# Exploring Trimethylaminuria: Genetics and Molecular Mechanisms, Epidemiology, and Emerging Therapeutic Strategies

**DOI:** 10.3390/biology13120961

**Published:** 2024-11-22

**Authors:** Antonina Sidoti, Rosalia D’Angelo, Andrea Castagnetti, Elisa Viciani, Concetta Scimone, Simona Alibrandi, Giuseppe Giannini

**Affiliations:** 1 Department of Biomedical and Dental Sciences and Morphofunctional Imaging, University of Messina, Via Consolare Valeria 1, 98125 Messina, Italy; asidoti@unime.it (A.S.); rdangelo@unime.it (R.D.); cscimone@unime.it (C.S.); 2 Wellmicro Srl, Via Antonio Canova, 30, 40138 Bologna, Italy; andrea.castagnetti@wellmicro.com (A.C.); elisa.viciani@wellmicro.com (E.V.); 3 Department of Biomolecular Strategies, Genetics, Cutting-Edge Therapies, I.E.ME.S.T., Via Michele Miraglia, 20, 90139 Palermo, Italy; 4 Alfasigma SpA, Via Pontina, km 30,400, 00071 Rome, Italy

**Keywords:** trimethylaminuria, gut microbiota dysbiosis, molecular genetics of FMO3

## Abstract

Trimethylaminuria (TMAU) is a rare metabolic syndrome caused by the accumulation of trimethylamine in the body, causing odor emissions similar to rotten fish in affected patients. This condition is determined by both genetic and environmental factors, especially gut dysbiosis. The multifactorial nature of this syndrome makes for a complex and multi-level diagnosis. To date, many aspects of this disease are still unclear. Recent research revealed the *FMO3* haplotypes’ role on the enzyme’s catalytic activity. This could explain why patients showing only combined polymorphisms or heterozygous causative variants also manifest the TMAU phenotype. In addition, another research hypothesized that the behavioral disturbances showed by patients may be linked to gut microbiota alterations. Our review considers current knowledge about TMAU, clarifying its molecular aspects, the therapeutic approaches used to limit this condition, and the new therapies that are under study.

## Simple Summary

Trimethylaminuria is a very rare and little-known metabolic syndrome. It is caused by the accumulation of a foul-smelling molecule, trimethylamine, which is excreted through biological fluids such as sweat, determining the symptoms in affected patients, which consist of odor emissions similar to rotten fish. TMA accumulation is determined by both genetic and environmental factors, especially gut dysbiosis. Although this syndrome is not physically disabling, patients have significant psychosocial problems which, in extreme cases, lead them to suicide. In this review, we collected the most updated data on the pathology, from diagnostic methods to therapeutics approaches, with the aim of encouraging the scientific community to study this syndrome for which there is still no definitive therapy.

## 1. Introduction

Trimethylaminuria (TMAU) is a rare metabolic disorder also known as “*Fish Odor Syndrome*”. It is little known, although literary citations dating back to 400 CE make it clear that this syndrome has early origins. Episodes of individuals showing the same symptoms have been described [1]. The first clinical case of TMAU was described in 1970 in the medical journal *Lancet* by Humbert et al., who first used the terms trimethylaminuria and fish-odor syndrome to describe a 6-year-old girl who intermittently had a fishy odor [2]. Two years later, Higgins et al. (1972) found a deficiency of trimethylamine oxidase by liver biopsy in the same patient [3].

TMAU is characterized by the accumulation and subsequent excretion of the aliphatic amine trimethylamine (TMA) through bodily fluids such as urine, sweat, saliva, vaginal secretions, and even breath. This is due to TMA’s chemical–physical properties, including its solubility in water and fat, as well as its ability to volatilize at room temperature [4]. The hallmark symptom reported by patients with TMAU is the emission of a pungent and unpleasant odor, like that of rotten fish.

Although in both healthy and affected individuals the most TMA production occurs in the gut by some microbiota species, it can also be synthesized in the oral cavity and in vaginal secretions. The main TMA precursors are trimethylamine N-oxide (TMAO), choline, L-carnitine, betaine, and ergothioneine, which are introduced through one’s diet. Foods such as fish, eggs, red meat, liver, legumes, peas, and peanuts are the main sources [5,6].

Studies on the metabolism of TMA precursors, particularly choline, were conducted as early as 1910 [7]. However, the metabolic pathways involved in TMA synthesis remained undiscovered for more than 100 years. Interest in understanding these mechanisms has grown proportionally to the increase in incidence of pathologies such as cardiovascular disease (CVD) and non-alcoholic fatty liver disease (NAFLD), which are associated with elevated levels of TMAO [8]. Despite this, studies carried out on a cohort of patients have shown that TMAO has no role as a possible marker of the pathologies mentioned above [9,10]. To date, the main enzymes involved in the metabolic pathways leading to TMA production have been identified as choline TMA lyase, carnitine monooxygenase, glycine-betaine reductase, and TMAO reductase [11]. However, the enzyme choline TMA lyase is currently the most studied, as it is known that choline’s conversion contributes significantly to TMA production.

In both healthy and affected patients, the TMA pathway is the same, but the TMA destiny is different. In healthy subjects, almost all the TMA produced is converted into the odorless oxidized form (TMAO). In affected patients, the TMA accumulates in the body, and its subsequent excretion results in the characteristic odor previously mentioned [4].

The causes of this accumulation can vary, leading to the identification of several forms of TMAU. The first is a genetic or primary form (TMAU1), which typically manifests during childhood due to mutations in the *FMO3* gene, resulting in a total or partial deficiency of the hepatic microsomal enzyme Flavin-containing monooxygenase 3 (FMO3) [12,13]. To date, this enzyme is the main one known to convert TMA into TMAO.

The secondary form (TMAU2), which generally appears in adulthood, is caused by environmental factors such as liver and/or kidney diseases and treatment with TMA precursors. However, in most cases, gut dysbiosis is the main risk factor [4,14]. TMA bacteria overproduction could lead to its accumulation due to saturation of the FMO3 enzyme.

Lastly, a transient form of trimethylaminuria exists, which can manifest intermittently depending on a patient’s physiological conditions [15,16]. In women, it can arise during menstruation due to hormonal alterations, also associated with the use of contraceptive pills, which may lead to lower expression of the FMO3 enzyme [17]. In newborns, it can also occur during the weaning period, which may result in the TMAU phenotype due to both an increase in TMA precursor levels introduced with the diet and the typical reduced expression of the FMO3 enzyme in this age group. Furthermore, intermittent forms can be triggered by intense physical activity, fever, and emotional stress [4,14].

Although trimethylaminuria is not a physically disabling disease, affected patients often experience significant psychosocial disturbances that compromise their daily activities. In most cases, patients become aware of their condition through rejection and unkind jokes from family, friends, co-workers, partners, and strangers. This leads to significant behavioral disorders such as anxiety, depression, and, in the most extreme cases, attempted suicide [18,19].

To date, it is unclear whether this disturbance is solely a consequence of the social discomfort caused by the emitted odor or if it could also be linked to the effects of metabolites originating from the gut dysbiosis affecting the patients. As detailed below, TMA may play a role in compromising the blood–brain barrier’s integrity (BBB).

Receiving a diagnosis is very important for the patient. Having confirmation that it is a medical condition and not a psychological belief is helpful for patients to cope with the disease. The first analysis recommended to patients is usually biochemical, consisting of determining the TMA and TMAO levels in urine. Subsequently, *FMO3* genetic screening is performed [20]. To date, unfortunately, many aspects of this disease remain poorly understood, making it difficult to advise patients on the correct therapeutic approach to follow. There are no effective treatments, only palliative measures to temporarily reduce the bad smell. Products such as chlorophyllin and activated charcoal are used to sequester the trimethylamine produced in the gut, riboflavin supplements are given to enhance residual FMO3 enzyme activity, and antibiotics (metronidazole, amoxicillin, and neomycin) are used to reduce the levels of TMA-producing gut bacteria [20]. In addition, patients are advised to avoid foods such as shellfish, eggs, meat, legumes, and mushrooms, which contain TMA precursors (choline, carnitine, betaine, TMAO).

## 2. Primary Trimethylaminuria (TMAU1)

Primary trimethylaminuria is the genetic form of this disease. It is caused by *FMO3* gene mutations that determine the partial or total malfunction of the flavin-containing monooxygenase 3 enzyme [12,13]. The FMO3 enzyme’s catalytic activity is, then, compromised, causing TMA accumulation and its subsequent excretion through biological fluids. Although TMAU is classified as a rare monogenic condition linked to *FMO3* gene mutations inherited in an autosomal recessive pattern, patients carrying combined polymorphisms or heterozygous causative variants can also manifest a mild TMAU phenotype [21]. The primary form has an early onset, coinciding with the weaning period. Newborns could manifest the TMAU phenotype because of both the increase in the TMA precursor levels introduced with the diet and the low expression of the *FMO3* gene.

### 2.1. FMO Gene Cluster

The *FMO3* gene belongs to an *FMO* gene cluster site on the long arm of chromosome 1. In humans, eleven *FMOs* have been identified, namely, five functional genes (*FMO1–5*) and six pseudogenes (*FMO6P–11P*). In detail, *FMO1* to *FMO4* genes and a pseudogene *FMO6P* belong to a gene cluster in the region q24.3. *FMO5* is located ~26 Mb closer to the centromere at 1q21.1. The second cluster contains only *FMO7P* to *FMO11P* pseudogenes at 1q24.2 [22].

*FMO1* is expressed in fetal liver but is silenced postnatally, remaining active in the kidneys and intestines [23]. *FMO2* is expressed in the lungs. *FMO4* is ubiquitously expressed, with preferential expression in the kidneys and the liver, and *FMO5* is expressed preferentially in the liver, the small intestine, and the stomach “https://www.uniprot.org/ (accessed on 20 July 2024)”. The structural organization of the *FMO3* gene will be discussed in the next paragraph.

#### *FMO3* Gene: Structural Organization

The *FMO3* gene maps on chromosome 1 at 1q24.3, is 27 kb long, and has nine exons, of which the first and part of the last one are non-coding [24]. *FMO3* is expressed in the human liver between birth and 2 years of age, increasing during childhood and adolescence, and, finally, reaching its maximum level in adults [25]. It codes for the FMO3 enzyme. *FMO3* mutations determine a FMO3 enzyme malfunction and, consequently, trimethylamine accumulation, determining the TMAU phenotype [12,13]. To date, more than 300 variants have been found in the gene, including more than 90 variants which have been associated with TMAU. Unfortunately, there are no global standard guidelines for classifying these variants, especially for polymorphisms. The genomic reference databases for diagnosis are mainly ClinVar, Lovd (https://databases.LOVd.nl/shared/genes/FMO3; accessed on 20 July 2024), and the Human Gene Mutation Database (HGMD^®^, (https://www.hgmd.cf.ac.uk/ac/index.php; accessed on 20 July 2024). According to the latter, 94 mutations within *FMO3* are known, classified as follows:

- 81 missense/nonsense mutations;
- 1 splicing mutation;
- 1 regulatory mutation;
- 7 small deletions;
- 2 small insertions;
- 1 small indels;
- 1 gross deletion.

Regarding the functional consequences on the disease’s phenotype, it is possible to further classify them in 59 disease-causing mutations (DMs), 18 mutations highly related to TMAU etiopathogenesis but not yet confirmed (DMs?), 13 functional polymorphisms with no reported disease association (FPs), 2 disease-associated polymorphisms (DPs), and 2 disease-associated polymorphisms with supporting functional evidence, e.g., by in vitro luciferase assay (DFPs).

### 2.2. Flavin-Containing Monooxygenase (FMO) Protein Family

The *FMO* gene cluster encodes microsomal, oxidoreductase, and NADPH-dependent enzymes belonging to the flavin-containing monooxygenase (FMO) family expressed in various organisms such as bacteria, fungi, plants, invertebrates, and vertebrates. The FMO was first studied in 1970 in pig liver microsomes by Dr. Daniel Ziegler at the University of Texas [26]. Later, in 1984, the FMO enzyme was purified from rabbit lung microsomes, demonstrating that multiple forms of FMO exist [27]. Despite their discovery over 40 years ago, the mammalian FMO structure has not yet been well described because X-ray crystallography could not be performed. Unlike the FMOs of yeast and bacteria, which are soluble, human FMOs are anchored to the endoplasmic reticulum membrane via the C-terminal domain. For these reasons, the dimeric tertiary structure of FMOs has only been studied in yeast and bacteria. Considering the percentage of sequence identity with human FMOs, 22% with yeasts and 31% with bacteria, it was demonstrated that the tertiary structures had similarities and all members were dimers composed of identical subunits [28,29]. Based on structural analysis reconstructions of ancestral sequences, human FMOs consist of two structural domains, a small domain that binds the NADPH cofactor, an extended domain that binds the FAD prosthetic group, and an active site. The NADPH and FAD cofactor binding domains are connected to each other by a double linker [30].

The FMO isoforms differ from one another in substrate specificity and differential tissue expression [25].

**Figure 1:**
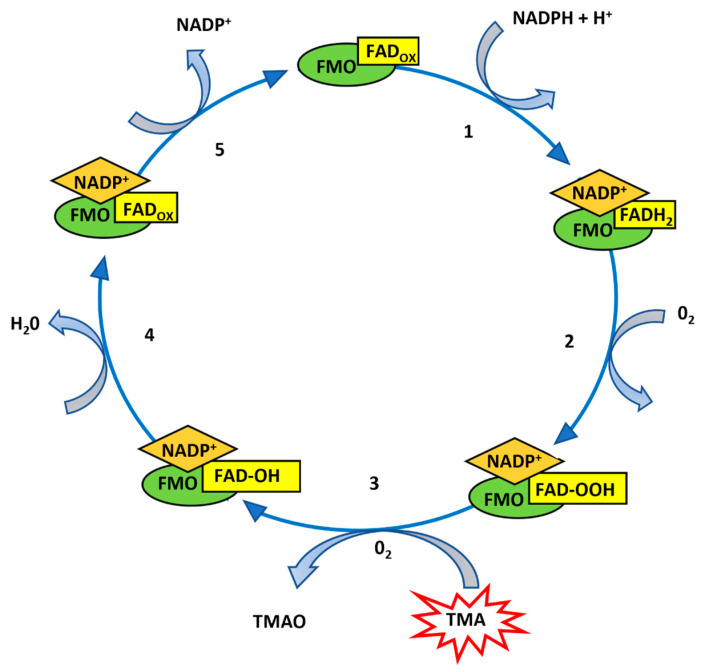
Catalytic cycle of FMO enzymes. This figure represents TMA oxidation by FMO enzymes, supported by NAD and FAD coenzymes.

Understanding of the catalytic mechanism of FMOs is based on the early work of Dan Ziegler and colleagues. FMOs contain FAD as a prosthetic group and, for catalysis, require NADPH as a cofactor and oxygen as a co-substrate. The substrate oxidation process takes place in two phases, one of which is faster than the other. In detail, the reaction begins when the NADPH cofactor binds to the FMO enzyme, reducing FAD to FADH_2_ (step 1). In step 2, molecular oxygen reacts with the NADP^+^–FADH_2_–enzyme complex, forming the 4a-hydroperoxyflavin compound (FMO-NADP^+^-FAD-OOH). In the following, slower step, step 3, the substrate is oxidized thanks to the oxygen nucleophilic attack released by the 4a-hydroperoxyflavin intermediate, which becomes C4a-hydroxyflavin (FMO-NADP^+^-FAD-OH). Finally (steps 4 and 5), water and NADP^+^ are released [31] (Figure 1).

#### FMO3 Chemical–Physical and Functional Properties

Flavin-containing monooxygenase 3 is a transmembrane protein of the endoplasmic reticulum containing 532 amino acid residues and a molecular mass of 60,047 Da [32]. It is mainly expressed in adult human liver but also in the skin [33], pancreas, cortex, and adrenal medulla [22]. Moreover, another variable is age. Age has been positively associated with enzyme abundance. This was significantly lower in the neonatal liver than in childhood (6–12 years) and adolescence (12–18 years) [25]. The FMO3 enzyme’s abundance decreases in women during menstruation as a response to female sex hormone variations, causing a decrease in the *FMO3* gene’s expression [15,16]. Regarding function, like the CYP3A4 enzyme, the FMO3 enzyme is involved in phase 1 detoxification reactions of drugs and endogenous substances [34]. Many of these are substrates of both enzymes. In recent years, in silico simulation models (Metasite) have been used to understand the enzyme–substrate interaction mode based on some parameters such as reactivity and the three-dimensional structure of the substrate [35]. The first is directly proportional to the nucleophilicity of the substrate, while the second has an important role because molecules with poorly exposed oxidation sites are less reactive [36].

### 2.3. Allele Frequency of the Most Common Polymorphisms

Since TMAU is a very rare syndrome, little is known about the disease’s incidence in the world population. An epidemiological study performed on the British population showed that heterozygous carrier incidence is 0.5–1% [37]. The first mutation that was identified in a trimethylaminuria patient was c.458C>T (p. Pro153Leu), which is one of the most common to date, together with c.913G>T (p. Glu305*), in individuals of European ethnicity. Instead, c.613C>T (p. Arg205Cys) and c.1498C>T (p. Arg500*) mutations are more diffused in the Japanese population [38,39]. D’Angelo et al. analyzed the frequency of four *FMO3* polymorphisms (Glu158Lys, Glu308Gly, Val257Met, and Gly475Asp) in Sicilian and Sardinian populations, highlighting that the Glu158Lys allele’s frequency is very similar in both populations, while Glu308Gly and Gly475Asp have a higher frequency in Sardinia. Conversely, Val257Met was not observed in Sardinia [40]. Moreover, the allele frequency of these polymorphisms, together with other variants (Asn285=, Ser147=, Asp132His, Val277Ala, Glu362Gln, and Gly180Val), was evaluated in African, Asian, and European ethnic groups. The polymorphic variant with the highest frequency in all considered groups was Glu158Lys. The Glu308Gly variant resulted also frequent in European and Asian populations but less common in Africa. In European and Asian populations, these two variants are found in cis on the same chromosome with a high frequency (20%). A third polymorphism, Val257Met, is more frequent in the Asian population but less frequent in European and African populations, but it does not influence the catalytic activity of the enzyme [39]. The Asp132His variant has a higher frequency in the African population, but it is very rare in Asian and European populations. In addition, the Val277Ala and Glu362Gln variants are frequent only in the African population, but their consequences on enzymatic activity have not yet been determined. The Gly180Val variant, found only in the European population, has no effect on enzymatic activity. All these polymorphisms individually have no effects on the enzyme’s catalytic activity but some of these cause its moderate decrease [38,40]. The role of *FMO3* haplotypes will be discussed in the next paragraph. Details on the frequencies of the mentioned polymorphisms are described in Table 1.

#### Could the *FMO3* Haplotypes Impair the Oxidative Activity of the Enzyme?

The genotype–phenotype association is one of the most discussed aspects of TMAU diagnosis. As already discussed, TMAU is transmitted in an autosomal recessive manner, and, referring to the diagnostic guidelines, the genetic test gives a positive result when causative homozygous mutations in *FMO3* are identified [38]. Despite this, patients showing combined polymorphisms or heterozygous causative variants often manifest the TMAU phenotype. The possible role of the *FMO3* haplotype in the catalytic activity of the enzyme was observed in Irish patients showing both mild and severe forms of TMAU. In detail, a correlation between the E158K/E308G haplotype, both in homozygous and heterozygous conditions, and the TMAU phenotype was observed. This association was further confirmed by the TMAO/TMA ratio in urine [41]. Subsequently, the role of other haplotypes, listed in Table 2, was investigated by Alibrandi et al. [42] A TMA/FMO3 docking prediction and an unbinding pathway study in patients who resulted positive for one or more causative and non-causative variants within *FMO3* were performed using different platforms and software.

This analysis highlighted that the studied haplotypes might cause enzymatic pocket narrowing, altering TMA transit through FMO3. Furthermore, such impairments could determine not only a different route for TMA, but also a reduced interaction time of the amine with the catalytic site of the enzyme.

## 3. Secondary Trimethylaminuria (TMAU2)

TMA accumulation also occurs in a secondary form. Unlike the primary genetic form, the secondary form is determined by environmental factors such as liver and kidney dysfunction, hormone therapy, and treatment with TMA precursors for Alzheimer’s and Huntington’s diseases [20,43]. In most cases, TMA accumulation is determined by gut dysbiosis conditions. The overproduction of bacterial TMA causes its accumulation; due to FMO3 enzyme saturation, TMA is then excreted through biological fluids, causing the characteristic and unpleasant rotten fish smell.

### TMA Production by Bacteria in the Human Gut

The gut microbiota consists of trillions of microorganisms, including bacteria, viruses, fungi, and other microbes, residing primarily in the large intestine, playing a crucial role in maintaining human health [44]. Intestinal bacteria live in a symbiotic interaction with the host, and, in healthy conditions, they can exert different functions, from the fermentation of the food that we eat to the stimulation of our immune system, the production of fundamental molecules like vitamins, and the release of neurochemicals like γ-Aminobutyric Acid (GABA), glutamate, tryptophan, serotonins, and catecholamines, which could affect the human neuronal system through metabolic pathways and neuronal stimuli which mediate communication between the intestine and the brain, an interconnection which has been called the gut–brain axis.

The microbiota is different in each ecological niche of our body and can be perturbed by environmental stimuli, health status, energy source availability, and pharmacological treatments; however, the bacterial component of the gut microbiota is composed mostly of six phyla: *Firmicutes*, *Bacteroidetes*, *Actinobacteria*, *Proteobacteria*, *Fusobacteria*, and *Verrucomicrobia*, with the first two phyla being the major types.

Gut microbes can use as growth substrate dietary components that escape absorption in the small intestine. In this way, the intestinal microbiota can exert its role in helping its human host harvest more nutrients from the diet by producing beneficial end products of microbial metabolism like short-chain fatty acids (SCFAs, e.g., butyrate) or otherwise beneficial metabolites which can be harmful for human health, like trimethylamine (TMA). The bacteria involved in TMA production in the gut, the metabolic pathways of bacterial production, and the catabolism of TMA have been previously reviewed [45].

Secondary TMAU, which is not determined by genetic factors, can be caused by an unbalanced intake of proteins from the diet or gut microbiota alterations; the latter is the most frequent cause for secondary TMAU.

Experiments performed by Smaranda Craciun and Emily P. Balskus identified a gene cluster encoding a pathway that involved the radical C-N bond cleavage of choline to generate TMA and acetaldehyde, a by-product of this metabolic process, in the choline-degrading sulfate-reducing bacterium *Desulfovibrio desulfuricans* [46]. This gene cluster contains *cutC* and *cutD* genes; the first one encodes a glycyl radical enzyme with choline trimethylamine-lyase activity, while the latter encodes a glycyl radical-activating protein, which acts as an activator on *CutC*. Both *CutC* and *CutD* are necessary for choline-derived TMA production [47]. There is a second major TMA synthesis pathway, which involves a two-component Rieske-type oxygenase/reductase (*CntA/B*) reaction that takes carnitine and its derivative γ-butyrobetaine as substrates [11]. Subsequently, another enzyme complex termed *YeaW/X*, which has close sequence similarity to *CntA/B*, was indicated as the key element of an additional major metabolic pathway involving carnitine utilization for TMA production [48]. Lately, it was discovered that the *YeaW/X* enzyme complex has substrate promiscuity for choline, carnitine γ-butyrobetaine, and betaine [49].

The functionality of the TMA-producing pathways has been demonstrated in all the microorganisms listed in Table 3. Instead, bacteria belonging to the phylum *Bacteroidetes* cannot produce TMA [50]. Gut bacteria are not the only ones who can produce TMA; even though this process takes place mainly in the gut, oral bacteria like *Streptococcus* can also produce TMA (Table 3).

Potential bacterial TMA production has been proposed by various researchers for other bacteria that have not been extensively tested for actual TMA production yet but possess the *CutC/D* gene cluster and/or the *CntA/B* or *YeaW/X* gene cluster. These bacterial species are listed in Table 4.

Long-term dietary habits can influence and modulate TMA production by the gut microbiota and antibiotic-like substances [60]. These molecules can be derived from food, plants, and herbs [61]. For example, allicin, present in crushed garlic cloves, supplemented to mice that had been administered carnitine modified their gut microbiota’s composition and decreased TMAO production, with a significant decrease in *Robinsoniella peoriensis* and an increase in *Clostridium* sp. in Culture jar-13, although more studies are required to assess whether these two bacterial species can be directly linked to TMAO level changes in the plasma of mice. Moreover, in human subjects showing high TMAO production, a 1-week-long raw garlic juice (which contains allicin) intervention reduced the TMAO-producing capacity of their intestinal microbiota and significantly enriched some anti-inflammatory gut commensal bacteria such as *Faecalibacterium prausnitzii* and *Akkermansia* spp.; these bacteria are not known TMA producers. Berberine, an isoquinoline alkaloid extracted from plants like *Coptis chinensis* and *Berberis vulgaris*, has been shown to attenuate TMA/TMAO production in mice fed a choline-enriched diet. Moreover, this choline treatment was associated with the increase in various gut bacteria and a TMA-producing bacterial genus such as *Desulfovibrio*, while berberine supplementation was not associated with the increase in known TMA-producing bacteria (*Bacteroideales* S24-7, *Alloprevotella*, and *Prevotellaceae* UCG-001) and showed a decrease in *cutC* and *cntA* gene abundance; of note, in anaerobic culture growth conditions, berberine displayed a strong suppression of the TMA-producing bacteria *Anaerococcus hydrogenalis* and *Clostridium sporogens* [62]. Resveratrol, a natural phytoalexin present in red wine, was able to suppress the TMA and TMAO levels in TMAO-induced atherosclerosis in *Apoe* knockout mice and could increase the proportion of non-TMA-producing *Lactobacillus* and *Bifidobacterium* bacterial genera.

The antibiotic-like activity of each compound should be well understood to assess whether there could be a risk of removing or inhibiting beneficial bacteria, but their use could be an interesting strategy to prevent TMA accumulation by TMA-producing gut microbiota.

## 4. TMAO Description and Diseases Association

Trimethylamine N-oxide (TMAO) is a colorless and odorless molecule, which is found in the tissues of various marine organisms and in bacteria, where it performs different functions [63]. In humans, TMAO is introduced through the diet and/or synthesized in the liver by the enzyme flavin-Containing Monooxygenase 3, which converts trimethylamine (TMA) into its oxidized form, TMAO. It is mostly contained in saltwater fish, shellfish, salmon, cod, tuna, swordfish, mackerel, sardines, and oily fish. About half of the TMAO introduced with one’s diet is not metabolized but is excreted in the urine, while the remaining 50% is converted into TMA by the TMAO reductase, a gut microbiota enzyme [50]. It has been documented that the TMAO levels in urine depend on both the fish species and the cooking method used. Freshwater fish intake does not seem to affect the TMAO levels in the urine. Fried fish consumption appears to be associated with an increase in TMAO values [64]. Recent studies have highlighted the role of TMAO in various pathologies such as type 2 diabetes mellitus, cardiovascular disease (CVD), atherosclerosis, colorectal cancer, and obesity. It is known that TMAO impairs reverse cholesterol transport, inducing lipid accumulation and foam cell formation in the arterial walls [50,65]. TMAO has also been found in cerebrospinal fluid, but it is not yet clear whether it is synthesized directly in the brain or whether it reaches it by crossing the blood–brain barrier. One of the most discussed topics, which also arouses conflicting opinions, concerns the association between fish consumption and CVD onset. Fish contains high concentrations of TMAO, which is known to be linked to the onset of CVD. At the same time, people who are affected by this disease are advised to consume fish rather than meat. This incongruity can be explained by considering the other compounds found in fish, which include polyunsaturated fatty acids, eicosapentaenoic acid, and docosahexanoic acid. Despite this, studies carried out in a cohort of patients have shown that TMAO has no role as a possible marker of the diseases mentioned above [9,10].

## 5. Diagnosis

Receiving a diagnosis is very important for the patient. Having the certainty that the reported symptoms are a real medical condition and not a psychological belief would help the patient live with the disease. Unfortunately, several factors lead to a delay in this diagnosis. For example, the bad odor is often not perceived by the patient or can be confused with other odors. Furthermore, the physicians are not always able to perceive it because the malodor can manifest itself intermittently. The first analysis recommended to the patient consists of the biochemical quantification of the levels of TMA and TMAO in the urine, but this check may not discriminate TMAU1 from TMAU2. *FMO3* genetic screening is useful to determine the TMAU1 condition.

### 5.1. Quantitative Analysis of Trimethylamine and Trimethylamine N-Oxide in Urine

The quantification of the TMA/TMAO ratio in the urine is very useful because it helps the clinician understand if an accumulation of TMA occurs. It is also essential to carefully evaluate the patient’s medical history because urinary tract infections [66], bacterial vaginosis [67], and advanced liver or kidney disease [68] could cause an accumulation of TMA, giving a false positive result, in which could also be the case in women during menstruation. The analysis involves the collection of urine samples on two separate occasions. Although testing can be performed under normal dietary conditions, it may help to consume a meal rich in choline (e.g., two eggs + 400 g “baked” [haricot] or soya beans) prior to testing [20]. TMA quantification is based on the TMAO/TMA ratio according to the formula TMAO/(TMAO + TMA) × 100. Ratios of 70–80% are classified as a mild phenotype, while ratios lower than 70% are classified as a severe phenotype. Various analytical techniques are used to determine TMA levels in urine, such as gas chromatography, liquid chromatography–mass spectrometry (LC/MS) [69,70], electrospray ionization tandem mass spectrometry (ESI-tandem-MS) [71], and proton nuclear magnetic resonance (H-NMR) spectroscopy [72]. The latter is the most used because it has more advantages. For example, TMA and TMAO can be detected simultaneously with great sensitivity, no metabolite extraction or separation is required, and measurements can be performed directly on urine samples.

### 5.2. FMO3 Genetic Screening

Genetic screening is performed to determine the causative variants in the *FMO3* gene, starting from DNA extracted from the peripheral blood. To date, more than 50 causative mutations are known. As already discussed, several studies have demonstrated the role of haplotypes in the folding of the FMO3 protein and, consequently, its catalytic activity [42]. Nevertheless, considering that this syndrome is transmitted in an autosomal recessive manner, referring to the diagnostic guidelines, genetic testing gives a positive result only when causative homozygous mutations in *FMO3* are identified [38].

### 5.3. Gut Microbiota Evaluation by Next-Generation Sequencing

A metagenomic analysis of the intestinal microbiota by next-generation sequencing (NGS) techniques might be a valid support for evaluating its contribution to the TMAU phenotype. The assessment of all the microbial components present in a fecal sample allows us to determine the potential dominance of TMA-producing bacteria in the gut ecosystem. The identification of an overabundance of TMA-producing bacteria would provide a new intervention target for TMAU phenotype amelioration.

## 6. Therapeutic Approach

To date, unfortunately, a definitive pharmacological treatment does not yet exist. Patients affected by both the primary and secondary forms of TMAU are offered only palliative treatments that temporarily reduce symptoms. The measures adopted by patients are different and aim to decrease the intake and/or the excretion of TMA through different strategies, which we can classify into the following four groups:

1. Precursor intake limitation;
2. Protonation of TMA;
3. Targeting of the gut metabolism;
4. Targeting of FMO3 enzyme.

### 6.1. Precursor Intake Limitation

One’s diet plays a fundamental role, given that TMA precursors (choline, carnitine, lecithin, ergothioneine, and TMAO) are contained in many foods, such as eggs, liver, legumes, peas, fish, peanuts, and others [45]. In addition, tannins and Brussels sprouts are not TMA precursors, but their consumption should be limited in these patients because they could inhibit the FMO3 enzyme’s catalytic activity. Choline performs many important functions for nerve and brain development in the fetus and in infants, as well as in the biosynthesis of essential phospholipids and acetylcholine neurotransmitters. Thus, a minimum amount of choline should be guaranteed, especially in pregnant or lactating women and children [20]. For this reason, patients with TMAU should be supported by a nutritionist recommending a balanced diet. Unfortunately, often patients do not rely on a specialist and start a do-it-yourself diet, causing potential deleterious effects.

### 6.2. TMA Capture

Various substances have a trapping effect towards TMA, according to the principle of electrostatic attraction. For example, negatively charged activated carbon attracts positive TMA by binding to it. It is known that taking 750 mg of activated charcoal 2 times a day for 10 days reduces urinary TMA levels in patients with TMAU. Another molecule used is copper chlorophyllin, which lowers the TMA levels by complexing with this molecule, and, unlike activated charcoal, the symptoms may reappear several weeks after the end of the treatment [73]. However, the use of these compounds is not recommended because their action mechanism is not specific to TMA, causing the capture of other important body metabolites. Furthermore, since TMA is a basic amino compound, the use of soaps and detergents with an acid pH could have a protonation effect, significantly reducing the odor being emitted [43]. Another approach to capture the TMA released from the body in patients with TMAU is to wear clothing made of fabrics capable of retaining protoned TMA. A patent was recently filed for a garment made of an odor-filtering material consisting of a layer of activated carbon, a first layer of fabric bonded to one side of the activated carbon layer, and a second layer of fabric bonded to the opposite side of the activated carbon layer. The thickness of the odor-filtering material is equal to or less than 0.8 mm; and the odor-filtering material is configured to be able to stretch by more than 20% in at least one direction. This fabric would also be suitable for use in the manufacturing of sports clothing. The patent number is WO2018210976A1.

### 6.3. Targeting the Gut Metabolism

Most of the interventions reported in the literature concern the inhibition of the metabolic processes that lead to TMA synthesis in the intestine. This goal can be achieved with different strategies.

#### 6.3.1. Antibiotic Treatment

Because TMA production from dietary precursors is dependent on its metabolism by the intestine, antibiotics have been assessed as a treatment for TMAU. Metronidazole, neomycin, and rifaximin are the main antibiotics administered [43,74].

Metronidazole. Reports on patients with TMAU receiving this antibiotic over a range of weeks showed variable results: urinary TMA was decreased by a factor of three in some patients, but this treatment did not show any effect in others. Improved outcomes were obtained when combining the treatment with dietary restrictions or another antibiotic [74].

Neomycin. Administration in mice has shown a reduction in the TMA levels in the urine; however, due to a non-permanent alteration of the microbiome, this antibiotic is often used together with metronidazole [74].

Rifaximin. Another antibiotic that has been explored for the treatment of TMAU is rifaximin. It has been reported to lower the TMA levels and generally be tolerated rather well, allowing for the localized targeting of enteric pathogens [75].

Although treatment with such antibiotics leads to a reduction in symptoms, the side effects outweigh the benefits of their use.

Their excessive administration could determine the emergence of antibiotic resistance and affect the bacterial infection. Furthermore, antibiotic treatment causes gut microbiota alterations and, consequentially, increases in the intestinal barrier’s permeability, leading to the onset of other diseases. Thus, their long-term use should be avoided [43].

#### 6.3.2. Administration of Probiotics

Probiotic treatments have recently grown as an alternative therapeutic approach to antibiotics. They have more beneficial effects and fewer side effects. Fecal microbial transplantation (FMT) has been performed in two patients with TMAU after metronidazole treatment. The attenuation of the fishy odor during the first 6 months after FMT was achieved only in one patient. Nevertheless, the bad smell came back after a year, suggesting that recurrent treatment would be necessary [76]. Unfortunately, despite repeated FMT administrations, the modulation of the gut microbiota community occurs very slowly, and this is one of the disadvantages of probiotics. In addition, probiotics’ low survival in the gastrointestinal tract represents another obstacle to overcome.

In addition, natural methanogenic *Archaea* of the human gut have been shown to reduce TMA with hydrogen. Although the underlying chemistry was described >40 years ago in the rumen of cows, delivering sufficient amounts of these oxygen-sensitive microorganisms to the gut remains an important hurdle. The technique was patented, but, so far, it has not been tested in humans.

#### 6.3.3. Other Probiotic Treatments

Several other probiotics have been tested for their ability to lower TMA levels, such as *Enterobacter aerogens* ZDY01, *Eubacterium limosum*, and members of the Clostridial clade XV family. However, so far, this has only been shown in vitro [77].

### 6.4. Targeting of the FMO3 Enzyme

Several studies have confirmed the modulatory action of some compounds on the catalytic activity of the FMO3 enzyme. For example, riboflavin is a precursor of the FAD prosthetic group of FMO3, thereby increasing its activity [78]. Conversely, Brussels sprouts and tannins have an inhibitory effect, especially if they are administered over a long period of time. Furthermore, the intake of drugs such as Clozapine, Deprenyl, and Ranitidine, metabolized by the FMO3 enzyme, should be avoided, because competing with TMA for the active site could worsen the TMAU phenotype [43].

## 7. Overview of TMA Lyase Inhibitors and Patents

As already fully described, there are various gut microbial enzymes that can produce TMA, but the main effort of researchers is currently focused on TMA lyase inhibitors. Various compounds have been investigated as potential inhibitors of TMA lyase, such as phytochemicals [79], choline analogs [80,81], stilbene-based derivatives [82], and flavonoids [83]. Additionally, 3,3-dimethyl-1-butanol (DMB) has shown efficacy in inhibiting microbial choline TMA lyase activity [84]. Some synthetic inhibitors, such as iodomethylcholine and fluoromethylcholine, have been found to be very potent and selective [85,86].

In conclusion, recent patent applications (Table 5) and research efforts on TMA lyase inhibitors highlight the growing interest in targeting microbial TMA production for therapeutic purposes.

By developing effective choline TMA lyase inhibitors, researchers offer a promising approach for modulating intestinal microbial activity to mitigate the impact of the production, and therefore dispersion, of TMA in patients suffering from TMAU.

## 8. Social Impact

Although TMAU is not life-threatening, affected patients often experience significant psychosocial disturbances that impact their daily activities [18,19]. Patients commonly realize that they are affected by this condition during interactions at school or work, where they may face comments about their personal hygiene such as “You should wash more often” or “There’s a smell in this room; let’s open the window”. These reactions can lead to embarrassment, feelings of inadequacy, discouragement, low self-esteem, isolation, and severe behavioral disorders such as anxiety, depression, and, in the most extreme cases, attempted suicide. The unpleasant odor negatively affects patients’ social relationships, particularly their career, personal life, love affairs, and education. They arrange their lives to accommodate this discomfort, often preferring jobs which minimize contact with others or strategically planning their workspace. Additionally, they exhibit obsessive–compulsive behaviors aimed at mitigating the unpleasant odor, such as taking frequent showers, using excessive amounts of perfume, or smoking cigarettes to mask the odor. These strategies are most commonly employed by these patients. Children are particularly affected by this condition, often being ridiculed and bullied by peers, leading to aggressive or disruptive behavior and poor academic performance.

## 9. Discussion and Conclusions

Even though it is still difficult to assess the exact prevalence of this ailment among the general population due to its infrequency, the severe form of TMAU affects 1 in 40,000 people [38]. Due to the low prevalence of this condition and scarce medical awareness, research efforts targeting the treatment of TMAU are still insufficient.

To date, it is still unknown whether the behavioral disorders that can afflict patients with TMAU are consequences of social reactions to the malodor or if they could be related to TMA accumulation. Recent in vitro and in vivo studies have shown that physiologically relevant concentrations of trimethylamine N-oxide (TMAO) enhance blood–brain barrier integrity and protect it from inflammatory insults, acting through the tight junction regulator annexin A1. In contrast, trimethylamine (TMA) impairs BBB function and disrupts tight junction integrity [87]. L. Donato et al. have developed the hypothesis that the gut–brain axis plays a crucial role in psychiatric disorders. An examination of the gut microbiota of patients with TMAU revealed that certain bacterial metabolites could cause the wide spectrum of psychiatric disorders observed in these patients. It is already known that many neurotransmitters such as serotonin, dopamine, and GABA are produced by the gut microbiota. Altered production, potentially due to gut dysbiosis, could induce biochemical alterations in the nervous system [88]. Thus, modulation of the gut microbiota via antibiotic-like molecules could be a promising approach, requiring more testing and a deeper understanding of the precise modifications which using these compounds could induce in the intestinal ecosystem.

This work collected the best resources regarding state-of-the-art knowledge around the TMAU condition, clarifying its origins, its molecular aspects, known medical treatments that can be used to limit this condition, and new therapies that are under study; however, there is still no definitive cure for this disease.

## Figures and Tables

**Table 1:** Frequencies of some polymorphisms in different ethnic groups.

Variant	SNP	Sicilian	Sardinian	Other European	African	Asian
c.394G>C [p. (Asp132His)]	rs12072582	˗	˗	0.00007	0.042	0.0001
c.441C>T [p. (Ser147=)]	rs1800822	˗	˗	0.053	0.080	0.190
c.539G>T [p. (Gly180Val)]	rs75904274	˗	˗	0.016	0.003	0.004
c.830T>C [p. (Val277Ala)]	rs2066530	˗	˗	0.00006	0.049	0.0001
c.1084G>C [p(Glu362Gln)]	rs2066532	˗	˗	0.00003	0.014	0
c.855C>T [p. (Asn285=)]	rs909530	˗	˗	0.252	0.440	0.284
c.472G>A [p. (Glu158Lys)]	rs2266782	0.3	0.3	0.420	0.464	0.283
c.923A>G [p. (Glu308Gly)]	rs2266780	0.1	0.2	0.202	0.035	0.096
c.769G>A [p. (Val257Met)]	rs1736557	0.2	0	0.065	0.035	0.132
c.1424G>A [p. (Gly475Asp)]	rs72549333	0.4	0.5	˗	˗	˗

**Table 2:** Phenotypic and genotypic characteristics of patients with TMAU.

ID	Age	Smell Description	*FMO3* Haplotypes
1	9	Rotten fish	Y331Stop
2	42	Rotten fish	E158K/P153L/P380Fs
3	48	Genital fishy odor, body garbage odor, and scalp acid/sulfur odor	P380Fs
4	70	Rotten fish	E308G/P380Fs
5	36	Rotten fish	E158K/P153L
6	71	Rotten fish	E158K/c.627+10 C>G
7	38	Rotten fish	V257M/c.627+10 C>G
8	45	Rotten fish	E158K/E308G/S147=
9	21	Rotten fish	E158K/R492W
10	73	Rotten fish	E158K/R238Q
11	51	Fish	E158K/G475D
12	20	Rotten fish	S147=/N285=/D141V/G180V

**Table 3:** Bacterial strains confirmed to degrade choline or carnitine into TMA.

Phylum	Microorganism	Reference	Choline	Carnitine
Proteobacteria	*Acinetobacter baumannii* (ATCC 19606)			+
	*Acinetobacter calcoaceticus* (ATCC 39647)			+
	*Acinetobacter calcoaceticus* 69/V			+
	*Citrobacter freundii* 4_7_47CFAA			+
	*Desulfovibrio alaskensis* G20		+	
	*Desulfovibrio desulfuricans* (ATCC 27774)		+	
	*Edwardsiella tarda* ATCC 23685		+	
	*Escherichia coli* DH10b	[48]		+
	*Escherichia coli* MS 200-1	[51]	+	
	*Escherichia coli* MS 69-1	[47]	+	
	*Escherichia coli* SE11	[11]		+
	*Escherichia fergusonii* (ATCC 35469)	[52]	+	
	*Klebsiella pneumoniae* (MSCL 535)	[53]		+
	*Klebsiella pneumoniae* (MSCL)	[54]	+	
	*Klebsiella* sp. MS 92-3		+	
	*Pelobacter acetylenicus*	[55]	+	
	*Pelobacter carbinolicus*	[56]	+	
	*Proteus mirabilis* (ATCC 29906)	[47]	+	
	*Proteus mirabilis* (DSM 4479)	[57]	+	
	*Proteus mirabilis* BB2000	[47]	+	
	*Proteus mirabilis* HI4320	[47]	+	
	*Proteus penneri* (ATCC 35198)	[52]	+	
	*Proteus vulgaris*	[58]	+	
	*Providencia rettgeri* (DSM 1131)	[52]	+	
	*Providencia rettgeri* (MSCL 730)	[54]		+
	*Serratia marcescens* (MSCL 1476)	[53]		+
Firmicutes	*Anaerococcus hydrogenalis* (DSM 7454)	[52]	+	
	*Clostridium asparagiforme* (DSM 15981)	[52]	+	
	*Clostridium citroniae* WAL-17108	[47]	+	
	*Clostridium hathewayi* (DSM 13749)	[52]	+	
	*Clostridium sporogenes* (ATCC 15579)	[52]	+	
	*Streptococcus dysgalactiae* (ATCC 12394)	[47]	+	
	*Streptococcus sanguis*	[59]	+	
Actinobacteria	*Olsenella uli* (DSM 7084)	[47]	+	

**Table 4:** Bacterial intestinal species that have the genetic potential to generate TMA. This table shows a list of potential TMA producers. * Only the *CutC* gene is present.

Phylum	Microorganism	*CutC/D* Gene Cluster	*CntA/B* Gene Cluster	*YeaW/X* Gene Cluster
Actinobacteria	*Collinsella tanakei*	+		
	*Corynebacterium ammoniagenes*		+	
Firmicutes	*Bacillus smithii*		+	
	*Paenibacillus* sp.		+	
	*Turicibacter* sp.	+ *		
Proteobacteria	*Acinetobacter junii*		+	
	*Arcobacter butzleri*		+	
	*Burkholderia oklahomensis*		+	
	*Citrobacter youngae*		+	
	*Enterobacter cancerogenus*		+	
	*Enterobacter cloacae*		+	
	*Escherichia albertii*		+	+
	*Neisseria macacae*		+	
	*Providencia alcalifaciens*	+		
	*Providencia rustigianii*	+		
	*Providencia stuartii*		+	+
	*Pseudomonas aeruginosa*		+	
	*Ralstonia* sp.		+	
	*Shigella* sp.		+	+
	*Vibrio furnissi*	+	+	
	*Yersinia pseudotuberculosis*		+	+

**Table 5:** Patent numbers related to TMAU. This table shows the up-to-date known patent numbers related to trimethylaminuria and associated diseases.

Patent Number	Title	Owner Companies	Indications
US-20180200291	Compositions comprising a zeolite and use thereof for the treatment of trimethylaminuria	NeuroSci Inc., Osceola, USA	Trimethylaminuria
WO-2014082773	Use of microorganisms for reducing the level of trimethylamine in a human body cavity, in particular for the treatment of trimethylaminuria or of bacterial vaginosis and the prevention of cardiovascular diseases	Université Clermont Auvergne (UCA), France	Bacterial vaginosis; cardiovascular disease; trimethylaminuria
JP-2021031483	The deodorizing liquid of the external composition for trimethylaminuria, and a trimethylamine smell	*Individual*	
GB-201706422	Therapeutic uses of flavin-containing monooxygenase 3	*Individual*	Trimethylaminuria
WO-2018033856	Transmembrane pH-gradient polymersomes and their use in the scavenging of ammonia and its methylated analogs	ETH Zürich- University in Zurich, Switzerland.	Hyperammonemia; trimethylaminuria
WO-2011072320	Effervescent L-carnitine-based composition	Jubilant Global Ltd., London, UK	Fatigue; flatulence; trimethylaminuria
US-20220125861	Reducing trimethylamine or trimethylamine-n oxide levels in a subject	Oregon State University (OSU), Corvallis, Oregon, USA	Bacterial vaginosis; cardiovascular disease; thrombosis; trimethylaminuria
US-11219387	Molecularly imprinted electrochemical sensors	University in Changchun, Jilin, China	Alzheimer’s disease; asthma; hyperglycemia; multiple sclerosis; trimethylaminuria
WO-2020118163	Decarboxylase inhibitors for treating Parkinson’s disease	Kintai Therapeutics Cambridge, MA, USA; Senda Biosciences Inc. Cambridge, MA, USA	Cardiovascular disease; diabetes mellitus; Parkinson’s disease; renal disease; trimethylaminuria
WO-2022151669	*Lactobacillus amylovorus* LAM1345 isolate, composition including the same and use thereof	Synbio Tech Inc. Kaohsiung City, Taiwan	Cancer; cardiovascular disease; diabetes mellitus; obesity; renal disease; trimethylaminuria
WO-2019070678	Methods for inhibiting conversion of choline to trimethylamine (TMA)	The Cleveland Clinic Foundation. Cleveland, Ohio. US; The Procter & Gamble Co. Cincinnati, Ohio. USA;	Angina; atherosclerosis; cardiovascular disease; diabetes mellitus; endocarditis; hyperlipidemia; hypertension; myocardial infarction; renal disease; stroke; trimethylaminuria
wO-2019070676	Methods for inhibiting conversion of choline to trimethylamine (TMA)	The Cleveland Clinic Foundation. Cleveland, Ohio. US; The Procter & Gamble Co. Cincinnati, Ohio. USA.	Angina; atherosclerosis; cardiovascular disease; diabetes mellitus; end-stage renal disease; heart arrhythmia; myocardial disease; obesity; renal disease; trimethylaminuria
wO-2019070677	Methods for inhibiting conversion of choline to trimethylamine (TMA)	The Cleveland Clinic Foundation. Cleveland, Ohio. USA. The Procter & Gamble Co. Cincinnati, Ohio. USA.	Atherosclerosis; cardiovascular disease; congestive heart failure; coronary artery disease; diabetes mellitus; end-stage renal disease; endocarditis; myocardial disease; renal disease; trimethylaminuria
WO-2020097151	Methods for inhibiting conversion of choline to trimethylamine (TMA)	The Cleveland Clinic Foundation. Cleveland, Ohio. USA. The Procter & Gamble Co. Cincinnati, Ohio. USA	Alzheimer’s disease; dementia; diabetes mellitus; end-stage renal disease; heart arrhythmia; insulin resistance; myocardial disease; non-alcoholic steatohepatitis; obesity; trimethylaminuria

## Data Availability

Not applicable.

## References

[1] Mitchell S.C. (1996). The fish-odor syndrome. Perspect. Biol. Med..

[2] Humbert J.A., Hammond K.B., Hathaway W.E. (1970). Trimethylaminuria: The fish-odour syndrome. Lancet.

[3] Higgins T., Chaykin S., Hammond K.B., Humbert J.R. (1972). Trimethylamine N-oxide synthesis: A human variant. Biochem. Med..

[4] Mitchell S.C., Smith R.L. (2001). Trimethylaminuria: The fish malodor syndrome. Drug Metab. Dispos..

[5] Simo C., Garcia-Canas V. (2020). Dietary bioactive ingredients to modulate the gut microbiota-derived metabolite TMAO. New opportunities for functional food development. Food Funct..

[6] Zhang A.Q., Mitchell S.C., Smith R.L. (1999). Dietary precursors of trimethylamine in man: A pilot study. Food Chem. Toxicol..

[7] Hayward H.R., Stadtman T.C. (1959). Anaerobic degradation of choline. I. Fermentation of choline by an anaerobic, cytochrome-producing bacterium, *Vibrio cholinicus* n. sp. J. Bacteriol..

[8] Falony G., Vieira-Silva S., Raes J. (2015). Microbiology Meets Big Data: The Case of Gut Microbiota-Derived Trimethylamine. Annu. Rev. Microbiol..

[9] Andraos S., Jones B., Lange K., Clifford S.A., Thorstensen E.B., Kerr J.A., Wake M., Saffery R., Burgner D.P., O’Sullivan J.M. (2021). Trimethylamine N-oxide (TMAO) Is not Associated with Cardiometabolic Phenotypes and Inflammatory Markers in Children and Adults. Curr. Dev. Nutr..

[10] Bhuiya J., Notsu Y., Kobayashi H., Shibly A.Z., Sheikh A.M., Okazaki R., Yamaguchi K., Nagai A., Nabika T., Abe T. (2023). Neither Trimethylamine-N-Oxide nor Trimethyllysine Is Associated with Atherosclerosis: A Cross-Sectional Study in Older Japanese Adults. Nutrients.

[11] Zhu Y., Jameson E., Crosatti M., Schafer H., Rajakumar K., Bugg T.D., Chen Y. (2014). Carnitine metabolism to trimethylamine by an unusual Rieske-type oxygenase from human microbiota. Proc. Natl. Acad. Sci. USA.

[12] Dolphin C.T., Janmohamed A., Smith R.L., Shephard E.A., Phillips I.R. (1997). Missense mutation in flavin-containing mono-oxygenase 3 gene, FMO3, underlies fish-odour syndrome. Nat. Genet..

[13] Treacy E.P., Akerman B.R., Chow L.M., Youil R., Bibeau C., Lin J., Bruce A.G., Knight M., Danks D.M., Cashman J.R. (1998). Mutations of the flavin-containing monooxygenase gene (*FMO3*) cause trimethylaminuria, a defect in detoxication. Hum. Mol. Genet..

[14] Mackay R.J., McEntyre C.J., Henderson C., Lever M., George P.M. (2011). Trimethylaminuria: Causes and diagnosis of a socially distressing condition. Clin. Biochem. Rev..

[15] Zhang A.Q., Mitchell S.C., Smith R.L. (1996). Exacerbation of symptoms of fish-odour syndrome during menstruation. Lancet.

[16] Shimizu M., Cashman J.R., Yamazaki H. (2007). Transient trimethylaminuria related to menstruation. BMC Med. Genet..

[17] Mayatepek E., Kohlmuller D. (1998). Transient trimethylaminuria in childhood. Acta Paediatr..

[18] Flaherty C.C., Phillips I.R., Janmohamed A., Shephard E.A. (2024). Living with trimethylaminuria and body and breath malodour: Personal perspectives. BMC Public Health.

[19] Roddy D., McCarthy P., Nerney D., Mulligan-Rabbitt J., Smith E., Treacy E.P. (2021). Impact of trimethylaminuria on daily psychosocial functioning. JIMD Rep..

[20] Phillips I.R., Shephard E.A., Adam M.P., Feldman J., Mirzaa G.M., Pagon R.A., Wallace S.E., Bean L.J.H., Gripp K.W., Amemiya A. (1993). Primary Trimethylaminuria. GeneReviews.

[21] Shimizu M., Allerston C.K., Shephard E.A., Yamazaki H., Phillips I.R. (2014). Relationships between flavin-containing mono-oxygenase 3 (*FMO3*) genotype and trimethylaminuria phenotype in a Japanese population. Br. J. Clin. Pharmacol..

[22] Hernandez D., Janmohamed A., Chandan P., Phillips I.R., Shephard E.A. (2004). Organization and evolution of the flavin-containing monooxygenase genes of human and mouse: Identification of novel gene and pseudogene clusters. Pharmacogenetics.

[23] Shephard E.A., Chandan P., Stevanovic-Walker M., Edwards M., Phillips I.R. (2007). Alternative promoters and repetitive DNA elements define the species-dependent tissue-specific expression of the *FMO1* genes of human and mouse. Biochem. J..

[24] Phillips I.R., Francois A., Shephard E. (2007). The Flavin-Containing Monoooxygenases (FMOs): Genetic Variation and its Consequences for the Metabolism of Therapeutic Drugs. Curr. Pharmacogenomics.

[25] Koukouritaki S.B., Simpson P., Yeung C.K., Rettie A.E., Hines R.N. (2002). Human hepatic flavin-containing monooxygenases 1 (*FMO1*) and 3 (*FMO3*) developmental expression. Pediatr. Res..

[26] Ziegler D.M., Poulsen L.L. (1978). Hepatic microsomal mixed-function amine oxidase. Methods Enzym..

[27] Williams D.E., Ziegler D.M., Nordin D.J., Hale S.E., Masters B.S. (1984). Rabbit lung flavin-containing monooxygenase is immunochemically and catalytically distinct from the liver enzyme. Biochem. Biophys. Res. Commun..

[28] Alfieri A., Malito E., Orru R., Fraaije M.W., Mattevi A. (2008). Revealing the moonlighting role of NADP in the structure of a flavin-containing monooxygenase. Proc. Natl. Acad. Sci. USA.

[29] Eswaramoorthy S., Bonanno J.B., Burley S.K., Swaminathan S. (2006). Mechanism of action of a flavin-containing monooxygenase. Proc. Natl. Acad. Sci. USA.

[30] Nicoll C.R., Bailleul G., Fiorentini F., Mascotti M.L., Fraaije M.W., Mattevi A. (2020). Ancestral-sequence reconstruction unveils the structural basis of function in mammalian FMOs. Nat. Struct. Mol. Biol..

[31] Ziegler D.M. (2002). An overview of the mechanism, substrate specificities, and structure of FMOs. Drug Metab. Rev..

[32] Dolphin C.T., Cullingford T.E., Shephard E.A., Smith R.L., Phillips I.R. (1996). Differential developmental and tissue-specific regulation of expression of the genes encoding three members of the flavin-containing monooxygenase family of man, FMO1, FMO3 and FM04. Eur. J. Biochem..

[33] Janmohamed A., Dolphin C.T., Phillips I.R., Shephard E.A. (2001). Quantification and cellular localization of expression in human skin of genes encoding flavin-containing monooxygenases and cytochromes P450. Biochem. Pharmacol..

[34] Phillips I.R., Shephard E.A. (2017). Drug metabolism by flavin-containing monooxygenases of human and mouse. Expert Opin. Drug Metab. Toxicol..

[35] Cruciani G., Baroni M., Benedetti P., Goracci L., Fortuna C.G. (2013). Exposition and reactivity optimization to predict sites of metabolism in chemicals. Drug Discov. Today Technol..

[36] Cruciani G., Valeri A., Goracci L., Pellegrino R.M., Buonerba F., Baroni M. (2014). Flavin monooxygenase metabolism: Why medicinal chemists should matter. J. Med. Chem..

[37] Al-Waiz M., Ayesh R., Mitchell S.C., Idle J.R., Smith R.L. (1987). A genetic polymorphism of the N-oxidation of trimethylamine in humans. Clin. Pharmacol. Ther..

[38] Shephard E.A., Treacy E.P., Phillips I.R. (2015). Clinical utility gene card for: Trimethylaminuria—Update 2014. Eur. J. Hum. Genet..

[39] Phillips I.R., Shephard E.A. (2020). Flavin-containing monooxygenase 3 (FMO3): Genetic variants and their consequences for drug metabolism and disease. Xenobiotica.

[40] D’Angelo R., Esposito T., Calabro M., Rinaldi C., Robledo R., Varriale B., Sidoti A. (2013). FMO3 allelic variants in Sicilian and Sardinian populations: Trimethylaminuria and absence of fish-like body odor. Gene.

[41] Doyle S., O’Byrne J.J., Nesbitt M., Murphy D.N., Abidin Z., Byrne N., Pastores G., Kirk R., Treacy E.P. (2019). The genetic and biochemical basis of trimethylaminuria in an Irish cohort. JIMD Rep..

[42] Schmidt A.C., Leroux J.C. (2020). Treatments of trimethylaminuria: Where we are and where we might be heading. Drug Discov. Today.

[43] Shreiner A.B., Kao J.Y., Young V.B. (2015). The gut microbiome in health and in disease. Curr. Opin. Gastroenterol..

[44] Fennema D., Phillips I.R., Shephard E.A. (2016). Trimethylamine and Trimethylamine N-Oxide, a Flavin-Containing Monooxygenase 3 (FMO3)-Mediated Host-Microbiome Metabolic Axis Implicated in Health and Disease. Drug Metab. Dispos..

[45] Craciun S., Balskus E.P. (2012). Microbial conversion of choline to trimethylamine requires a glycyl radical enzyme. Proc. Natl. Acad. Sci. USA.

[46] Martinez-del Campo A., Bodea S., Hamer H.A., Marks J.A., Haiser H.J., Turnbaugh P.J., Balskus E.P. (2015). Characterization and detection of a widely distributed gene cluster that predicts anaerobic choline utilization by human gut bacteria. mBio.

[47] Koeth R.A., Levison B.S., Culley M.K., Buffa J.A., Wang Z., Gregory J.C., Org E., Wu Y., Li L., Smith J.D. (2014). gamma-Butyrobetaine is a proatherogenic intermediate in gut microbial metabolism of L-carnitine to TMAO. Cell Metab..

[48] Zeisel S.H., Warrier M. (2017). Trimethylamine N-Oxide, the Microbiome, and Heart and Kidney Disease. Annu. Rev. Nutr..

[49] Janeiro M.H., Ramirez M.J., Milagro F.I., Martinez J.A., Solas M. (2018). Implication of Trimethylamine N-Oxide (TMAO) in Disease: Potential Biomarker or New Therapeutic Target. Nutrients.

[50] Romano K.A., Martinez-Del Campo A., Kasahara K., Chittim C.L., Vivas E.I., Amador-Noguez D., Balskus E.P., Rey F.E. (2017). Metabolic, Epigenetic, and Transgenerational Effects of Gut Bacterial Choline Consumption. Cell Host Microbe.

[51] Romano K.A., Vivas E.I., Amador-Noguez D., Rey F.E. (2015). Intestinal microbiota composition modulates choline bioavailability from diet and accumulation of the proatherogenic metabolite trimethylamine-N-oxide. mBio.

[52] Kalnins G., Sevostjanovs E., Hartmane D., Grinberga S., Tars K. (2018). CntA oxygenase substrate profile comparison and oxygen dependency of TMA production in Providencia rettgeri. J. Basic Microbiol..

[53] Kalnins G., Kuka J., Grinberga S., Makrecka-Kuka M., Liepinsh E., Dambrova M., Tars K. (2015). Structure and Function of CutC Choline Lyase from Human Microbiota Bacterium *Klebsiella pneumoniae*. J. Biol. Chem..

[54] Schink B. (1985). Fermentation of Acetylene by an Obligate Anaerobe, *Pelobacter acetylenicus* sp.nov. Arch. Microbiol..

[55] Aklujkar M., Haveman S.A., DiDonato R., Chertkov O., Han C.S., Land M.L., Brown P., Lovley D.R. (2012). The genome of *Pelobacter carbinolicus* reveals surprising metabolic capabilities and physiological features. BMC Genom..

[56] Jameson E., Fu T., Brown I.R., Paszkiewicz K., Purdy K.J., Frank S., Chen Y. (2016). Anaerobic choline metabolism in microcompartments promotes growth and swarming of *Proteus mirabilis*. Environ. Microbiol..

[57] Seim H., Löster H., Claus R., Kleber H.-P., Strack E. (1982). Formation of γ-butyrobetaine and trimethylamine from quaternary ammonium compounds structure-related to l-carnitine and choline by *Proteus vulgaris*. FEMS Microbiol. Lett..

[58] Chao C.K., Zeisel S.H. (1990). Formation of trimethylamine from dietary choline by *Streptococcus sanguis I*, which colonizes the mouth. J. Nutr. Biochem..

[59] Panyod S., Wu W.K., Chen C.C., Wu M.S., Ho C.T., Sheen L.Y. (2023). Modulation of gut microbiota by foods and herbs to prevent cardiovascular diseases. J. Tradit. Complement. Med..

[60] Waksman S.A. (1947). What is an antibiotic or an antibiotic substance?. Mycologia.

[61] Li X., Su C., Jiang Z., Yang Y., Zhang Y., Yang M., Zhang X., Du Y., Zhang J., Wang L. (2021). Berberine attenuates choline-induced atherosclerosis by inhibiting trimethylamine and trimethylamine-N-oxide production via manipulating the gut microbiome. NPJ Biofilms Microbiomes.

[62] Velasquez M.T., Ramezani A., Manal A., Raj D.S. (2016). Trimethylamine N-Oxide: The Good, the Bad and the Unknown. Toxins.

[63] Lombardo M., Aulisa G., Marcon D., Rizzo G., Tarsisano M.G., Di Renzo L., Federici M., Caprio M., De Lorenzo A. (2021). Association of Urinary and Plasma Levels of Trimethylamine N-Oxide (TMAO) with Foods. Nutrients.

[64] Dalla Via A., Gargari G., Taverniti V., Rondini G., Velardi I., Gambaro V., Visconti G.L., De Vitis V., Gardana C., Ragg E. (2019). Urinary TMAO Levels Are Associated with the Taxonomic Composition of the Gut Microbiota and with the Choline TMA-Lyase Gene (*cutC*) Harbored by Enterobacteriaceae. Nutrients.

[65] Lam C.W., Law C.Y., Sze K.H., To K.K. (2015). Quantitative metabolomics of urine for rapid etiological diagnosis of urinary tract infection: Evaluation of a microbial-mammalian co-metabolite as a diagnostic biomarker. Clin. Chim. Acta.

[66] Wolrath H., Boren H., Hallen A., Forsum U. (2002). Trimethylamine content in vaginal secretion and its relation to bacterial vaginosis. APMIS.

[67] Maksymiuk K.M., Szudzik M., Gawrys-Kopczynska M., Onyszkiewicz M., Samborowska E., Mogilnicka I., Ufnal M. (2022). Trimethylamine, a gut bacteria metabolite and air pollutant, increases blood pressure and markers of kidney damage including proteinuria and KIM-1 in rats. J. Transl. Med..

[68] Jia X., Osborn L.J., Wang Z. (2020). Simultaneous Measurement of Urinary Trimethylamine (TMA) and Trimethylamine N-Oxide (TMAO) by Liquid Chromatography-Mass Spectrometry. Molecules.

[69] Schlesinger P. (1979). An improved gas--liquid chromatographic method for analysis of trimethylamine in urine. Anal. Biochem..

[70] Mamer O.A., Choiniere L., Treacy E.P. (1999). Measurement of trimethylamine and trimethylamine N-oxide independently in urine by fast atom bombardment mass spectrometry. Anal. Biochem..

[71] Matsushita K., Kato K., Ohsaka A., Kanazawa M., Aizawa K. (1989). A simple and rapid method for detecting trimethylamine in human urine by proton NMR. Physiol. Chem. Phys. Med. NMR.

[72] Alibrandi S., Nicita F., Donato L., Scimone C., Rinaldi C., D’Angelo R., Sidoti A. (2021). Adaptive Modelling of Mutated FMO3 Enzyme Could Unveil Unexplored Scenarios Linking Variant Haplotypes to TMAU Phenotypes. Molecules.

[73] Yamazaki H., Fujieda M., Togashi M., Saito T., Preti G., Cashman J.R., Kamataki T. (2004). Effects of the dietary supplements, activated charcoal and copper chlorophyllin, on urinary excretion of trimethylamine in Japanese trimethylaminuria patients. Life Sci..

[74] Chalmers R.A., Bain M.D., Michelakakis H., Zschocke J., Iles R.A. (2006). Diagnosis and management of trimethylaminuria (FMO3 deficiency) in children. J. Inherit. Metab. Dis..

[75] Descombe J.J., Dubourg D., Picard M., Palazzini E. (1994). Pharmacokinetic study of rifaximin after oral administration in healthy volunteers. Int. J. Clin. Pharmacol. Res..

[76] Lahtinen P., Mattila E., Anttila V.J., Tillonen J., Teittinen M., Nevalainen P., Salminen S., Satokari R., Arkkila P. (2017). Faecal microbiota transplantation in patients with Clostridium difficile and significant comorbidities as well as in patients with new indications: A case series. World J. Gastroenterol..

[77] Qiu L., Yang D., Tao X., Yu J., Xiong H., Wei H. (2017). Enterobacter aerogenes ZDY01 Attenuates Choline-Induced Trimethylamine N-Oxide Levels by Remodeling Gut Microbiota in Mice. J. Microbiol. Biotechnol..

[78] Manning N.J., Allen E.K., Kirk R.J., Sharrard M.J., Smith E.J. (2012). Riboflavin-responsive trimethylaminuria in a patient with homocystinuria on betaine therapy. JIMD Rep..

[79] Iglesias-Carres L., Essenmacher L.A., Racine K.C., Neilson A.P. (2021). Development of a High-Throughput Method to Study the Inhibitory Effect of Phytochemicals on Trimethylamine Formation. Nutrients.

[80] Gabr M., Swiderek K. (2020). Discovery of a Histidine-Based Scaffold as an Inhibitor of Gut Microbial Choline Trimethylamine-Lyase. ChemMedChem.

[81] Wang Z., Roberts A.B., Buffa J.A., Levison B.S., Zhu W., Org E., Gu X., Huang Y., Zamanian-Daryoush M., Culley M.K. (2015). Non-lethal Inhibition of Gut Microbial Trimethylamine Production for the Treatment of Atherosclerosis. Cell.

[82] Li J., Huang P., Cheng W., Niu Q. (2023). Stilbene-based derivatives as potential inhibitors of trimethylamine (TMA)-lyase affect gut microbiota in coronary heart disease. Food Sci. Nutr..

[83] Zhou P., Zhao X.N., Ma Y.Y., Tang T.J., Wang S.S., Wang L., Huang J.L. (2022). Virtual screening analysis of natural flavonoids as trimethylamine (TMA)-lyase inhibitors for coronary heart disease. J. Food Biochem..

[84] Jonsson A.L., Backhed F. (2015). Drug the Bug!. Cell.

[85] Roberts A.B., Gu X., Buffa J.A., Hurd A.G., Wang Z., Zhu W., Gupta N., Skye S.M., Cody D.B., Levison B.S. (2018). Development of a gut microbe-targeted nonlethal therapeutic to inhibit thrombosis potential. Nat. Med..

[86] Helsley R.N., Miyata T., Kadam A., Varadharajan V., Sangwan N., Huang E.C., Banerjee R., Brown A.L., Fung K.K., Massey W.J. (2022). Gut microbial trimethylamine is elevated in alcohol-associated hepatitis and contributes to ethanol-induced liver injury in mice. Elife.

[87] Hoyles L., Pontifex M.G., Rodriguez-Ramiro I., Anis-Alavi M.A., Jelane K.S., Snelling T., Solito E., Fonseca S., Carvalho A.L., Carding S.R. (2021). Regulation of blood-brain barrier integrity by microbiome-associated methylamines and cognition by trimethylamine N-oxide. Microbiome.

[88] Donato L., Alibrandi S., Scimone C., Castagnetti A., Rao G., Sidoti A., D’Angelo R. (2021). Gut-Brain Axis Cross-Talk and Limbic Disorders as Biological Basis of Secondary TMAU. J. Pers. Med..

